# Effects of Smoking and Physical Activity on the Pulmonary Function of Young University Nursing Students in Cáceres (Spain)

**DOI:** 10.1097/jnr.0000000000000322

**Published:** 2019-09-20

**Authors:** Sergio RICO-MARTÍN, Jorge M. DE NICOLÁS-JIMÉNEZ, Mariana MARTÍNEZ-ÁLVAREZ, Sergio CORDOVILLA-GUARDIA, Esperanza SANTANO-MOGENA, Julián F. CALDERÓN-GARCÍA

**Affiliations:** 1 PhD, RN, Associate Professor, Department of Nursing, Nursing and Occupational Therapy College, University of Extremadura, Cáceres, Spain; 2 PhD, MD, Primary Attention Doctor, Zona Centro Health Center, Extremadura Health Service, Cáceres, Spain; 3 PhD, RN, Associate Professor, Department of Nursing, Faculty of Medicine, University of Extremadura, Badajoz, Spain; 4 PhD, RN, Associate Professor, Department of Nursing, Nursing and Occupational Therapy College, University of Extremadura, Cáceres, Spain; 5 RN, Teaching Assistant, Department of Nursing, Nursing and Occupational Therapy College, University of Extremadura, Cáceres, Spain; 6 PhD, RN, Professor, Department of Nursing, Nursing and Occupational Therapy College, University of Extremadura, Cáceres, Spain.

**Keywords:** pulmonary function, smokers, physical activity, young, nursing students

## Abstract

**Background:**

The simultaneous effect of physical activity (PA) and smoking on pulmonary function in young people remains unclear.

**Propose:**

The aim of this study was to determine the influence of smoking and PA on pulmonary function in young university students in Cáceres, Spain.

**Methods:**

A sample of 120 young nursing students was studied (60 smokers and 60 nonsmokers). All subjects underwent spirometry with a COPD-6 portable device, and their level of PA was quantified using the International Physical Activity Questionnaire. The influence of PA and smoking on pulmonary function was determined by comparing hypotheses.

**Results:**

Significant differences were observed between smokers and nonsmokers in terms of percent forced expiratory volume in 1 second, percent forced expiratory volume in 6 seconds, lung age, and the difference between lung age and chronological age (LA–CA) in those who practiced mild PA. In the subjects who performed moderate and vigorous PA, these differences were not noted. In the intragroup analysis, significant differences were observed in smokers in terms of percent forced expiratory volume in 1 second, percent forced expiratory volume in 6 seconds, lung age, and LA–CA; however, in the control group, differences were only observed in terms of lung age and LA–CA. These findings were confirmed in the multivariate analysis.

**Conclusions/Implications for Practice:**

Our findings confirmed a deterioration in pulmonary function in smokers who did not perform moderate or vigorous PA. The level of PA performed was positively related to pulmonary function in smokers, whereas in nonsmokers, improvements were only significant in LA–CA.

## Introduction

Although the incidence of smoking has decreased during the past few years (Ng et al., 2014), smoking remains one of the main risk factors for preventable morbidity and mortality in the world (Lim et al., 2012). According to the National Institute of Statistics of Spain, it is estimated that 22.98% of the population smokes daily, with a somewhat lower incidence (18.51%) in young people under 24 years old. Most smokers begin at the age of 14 years, and a large number of these individuals already consume cigarettes on a regular basis when they reach the age of majority. This age range represents a vital stage in which there is a consolidation of personality that leads to the maintenance of healthy habits and lifestyles throughout life.

Tobacco smoke affects the airways and lungs of young people who smoke actively or are passively exposed on a regular basis and is associated with adverse health problems in the upper and lower respiratory tract, including higher rates of asthma and reduced pulmonary function (Gibbs, Collaco, & McGrath-Morrow, 2016). In healthy adults, tobacco smoke has been shown to be the main cause of chronic obstructive pulmonary disease (COPD) and chronic respiratory symptoms such as chronic cough, increased production of phlegm, wheezing, and shortness of breath (Forey, Thornton, & Lee, 2011). In addition, tobacco smoke causes a decrease in pulmonary function (Simmons et al., 2005).

The medical literature clearly shows the beneficial effects of physical activity (PA) on health, reducing the risk of morbidity and mortality of many diseases and helping maintain and/or improve individuals' independence and functional capacities (Fogelholm, 2010). Cohort studies have indicated that the regular practice of PA may be effective in both the prevention and treatment of chronic respiratory diseases, including asthma and COPD (Lahti, Holstila, Lahelma, & Rahkonen, 2014). Furthermore, a positive relationship has been confirmed between physical exercise and spirometric parameters (Gimeno-Santos et al., 2014; Paulo, Petrica, & Martins, 2013), although some studies have not confirmed this relationship (Smith et al., 2016).

Because smoking and PA are largely incongruent behaviors (Kaczynski, Manske, Mannell, & Grewal, 2008) and the simultaneous effect on pulmonary function in young people remains unclear (Campbell Jenkins et al., 2014; Holmen, Barrett-Connor, Clausen, Holmen, & Bjermer, 2002; Michalak, Gatkiewicz, Pawlicka-Lisowska, & Poziomska-Piatkowska, 2012), the objective of this study was to determine the influence of smoking and PA on pulmonary function in young university students (aged 18–24 years) in Cáceres, Spain. The hypothesis is that PA may have a beneficial effect on lung capacity in young people who smoke.

## Methods

### Design and Population

**Figure 1.**
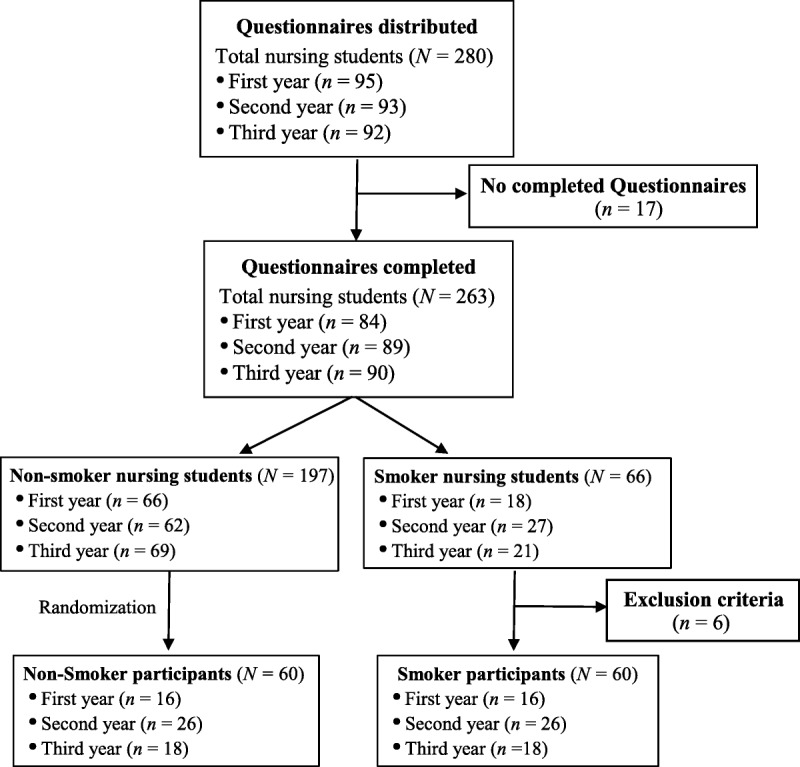
Sample selection process.

This cross-sectional, descriptive, association study was conducted on a sample of 120 healthy young people (94 women and 26 men) between 18 and 24 years old. The recruitment was performed during the months of March and April 2017. The subjects were first-, second-, and third-year nursing students from the Cáceres university campus. Figure 1 illustrates the sample selection process. Before the inclusion of participants, a questionnaire was conducted to determine the number of students who smoked on a regular basis (*n* = 66). The sample was categorized into two groups of 60 subjects according to whether they were current smokers or had never smoked. The group of current smokers (*n* = 60) accounted for 91% of the total active smokers. Nonsmoking subjects were randomly selected from the total of nonsmoking students. All of the participants were informed of the nature of the investigation and gave their written consent to participate. The study was designed in accordance with the Declaration of Helsinki, and the protocol was approved by the local ethics committee (No. 18000574).

The exclusion criteria in this study were as follows: (a) presence of asthma, (b) presence of catarrh or constipation at the time of the study, (c) presence of diseases that affect lung capacity, (d) presence of thoracic deformity, and (e) being a former smoker.

Data were collected from all of the participants on age, gender, and disease history. A physical examination was conducted to determine weight, height, body mass index, heart rate, and arterial oxygen saturation. For smokers, the following variables were recorded: age when they started smoking (years), how long they had smoked (years), number of cigarettes consumed daily, pack-years, amount of time between getting up in the morning and the first cigarette (minutes), and type of cigarette usually consumed (rolled or manufactured). The Fagerström test, which is used worldwide to assess the degree of physical dependence on tobacco smoking, was used to assess level of nicotine addiction (Fagerstrom & Schneider, 1989). This test has six items, with a total score ranging from 0 to 10. Scores were categorized into low dependence (≤ 4), medium dependence (5–6), and high dependence (≥ 7). The Spanish version was previously validated, and the reliability coefficient was .66 (Becoña & Vázquez, 1998). The Richmond test was used to determine the degree of motivation to stop smoking (Richmond, Kehoe, & Webster, 1993). This test has four items, with a total possible score ranging from 0 to 10. Motivation is considered low when the score is between 0 and 5, moderate when the score is between 6 and 8, and high when the score is 9 or 10.

### Pulmonary Function Assessment

Each participant underwent forced spirometry with a COPD-6 portable spirometer (Vitalograph). The diagnostic utility of this device was previously validated (Represas Represas et al., 2010) and has been recently used in several studies to determine pulmonary function (Figueira Gonçalves et al., 2017; Kjeldgaard, Lykkegaard, Spillemose, & Ulrik, 2017). The recommendations of the Spanish Society of Pneumology and Thoracic Surgery were followed for the spirometry tests conducted in this study (García-Río et al., 2013). Three maneuvers were performed for each patient, which met the criteria of acceptability and reproducibility, and the best device was selected for each parameter. Nasal clamps were not used, and no bronchodilator test was performed. The values of percent forced expiratory volume in 1 second (FEV_1_%), percent forced expiratory volume in 6 seconds (FEV_6_%), and FEV_1_/FEV_6_ (%) adjusted for age, gender, height, and lung age were measured using the device. The difference between lung age and chronological age (LA–CA) was calculated for each of the participants. The tests were performed by the same operator, who had been trained in the same laboratory (the Nursing and Occupational Therapy College of Cáceres).

### Assessment of Physical Activity

The level of PA was quantified using the International Physical Activity Questionnaire-Short Form (Craig et al., 2003). This questionnaire consists of seven questions and is used to measure the intensity of PA performed over a 1-week period in different aspects of people's lives (work, household tasks, transportation, and leisure). This questionnaire classifies PA into three categories: mild, moderate, and vigorous. The International Physical Activity Questionnaire-Short Form questionnaire has shown adequate validity for use with Spanish university students (Rodriguez-Muñoz, Corella, Abarca-Sos, & Zaragoza, 2017).

### Statistical Analysis

All values were expressed in terms of mean and standard deviation, medians (interquartile ranges), frequency, and percentage. Before applying the standard tests, the Kolmogorov–Smirnov test of goodness of fit was performed to verify whether the data had a normal distribution, and the Levene test was used to determine if the distribution of the samples assumed equal variances. The comparative study of smokers and nonsmokers was performed with the *t* test for independent samples when the data were parametric, and the Mann–Whitney *U* test was used when the data were not parametric. The groups were stratified according to the level of PA performed, and the Kruskal–Wallis test was used for intragroup comparisons. The Pearson chi-square test was used to compare categorical variables.

A multivariate logistic regression analysis was performed to measure the association between independent variables and the dependent variable. The quartile with the worst spirometric values provided the cutoff points for FEV_1_% (≤ 92%), FEV_6_% (≤ 90%), FEV_1_%/FEV_6_% (≤ 100%), and lung year (≥ 33 years). The cutoff point for LA–CA was ≥ 1 year, that is, having a pulmonary age that is higher than chronological age. Two adjusted models were used to perform the multivariate analysis. Model 1 was adjusted by smoking, PA level, years, gender, body mass index, and packs consumed per year. Afterward, the participants were stratified into two groups (smokers and nonsmokers). A multivariate analysis adjusted by PA level, years, gender, body mass index, and packs consumed per year was performed. Data were expressed as odds ratio (*OR*) and 95% confidence interval (CI).

The significance threshold was *p* < .05 for all the statistical tests. Statistical analysis was performed using IBM SPSS Version 24 (IBM Corporation, Armonk, NY, USA).

## Results

This study included 120 young nursing students (22% male) between 19 and 24 years old as participants. The main characteristics of the smoking and nonsmoking participants are shown in Table 1. Vigorous PA was more frequent in the nonsmoker group than the smoker group (33% vs. 18%, respectively). No significant differences were found in any of the variables analyzed.

**TABLE 1.**
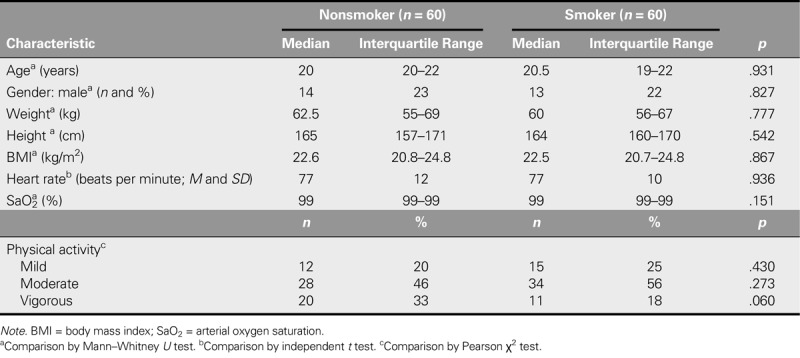
Baseline Characteristics of the Groups

Table 2 shows the data related to consumption of tobacco in the smoking group. The average time smoking was 5 ± 3 years, and the average proportion of packs consumed per year was 2 ± 2. Regarding nicotine dependence, most showed low dependence (78%), and most had a low motivation to quit (45%).

**TABLE 2.**
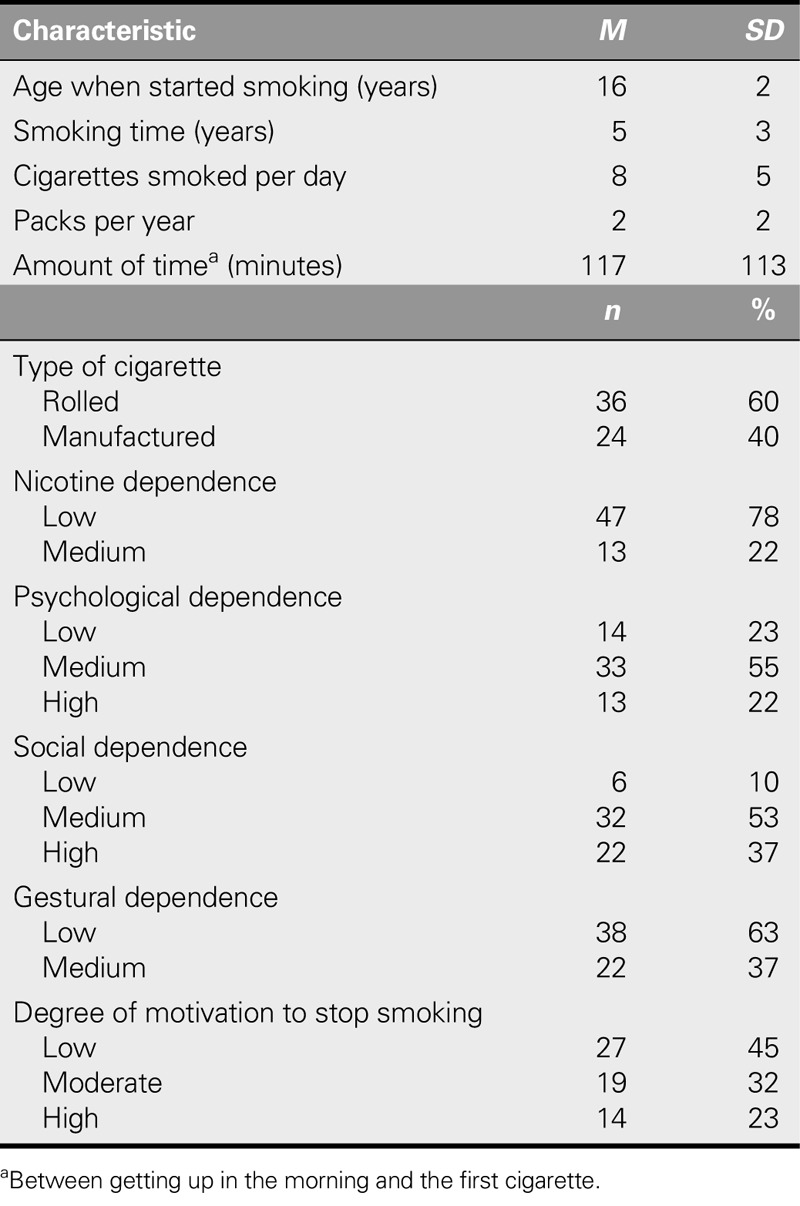
Characteristics Related to the Consumption of Tobacco in the Smoking Group (*N* = 60)

Table 3 shows the results of the comparative study between the groups regarding spirometry variables. Significant differences were observed between both groups (*p* < .05), with the group of nonsmokers obtaining better results in FEV_1_%, lung age, and LA–CA. Table 3 also shows the relationship between pulmonary function in both groups, stratified according to PA level. Significant differences (*p* < .05) were observed in the spirometric values of FEV_1_%, FEV_6_%, lung age, and LA–CA in those who practiced mild PA. In the nonsmoking group, with the exception of FEV_6_%, these differences were not detected in those participants who performed moderate or vigorous PA, even with better spirometric values. In the intragroup analysis, significant differences were observed in FEV_1_%, FEV_6_%, lung age, and LA–CA in smokers. However, in the control group, differences were only found in lung age and LA–CA. In the smoking group, these differences were mainly seen between the mild PA group and the moderate PA group and between the mild PA group and the vigorous PA group, whereas differences in the group of nonsmokers existed between the mild PA group and the vigorous PA group and between the vigorous PA group and the moderate PA group.

**TABLE 3.**
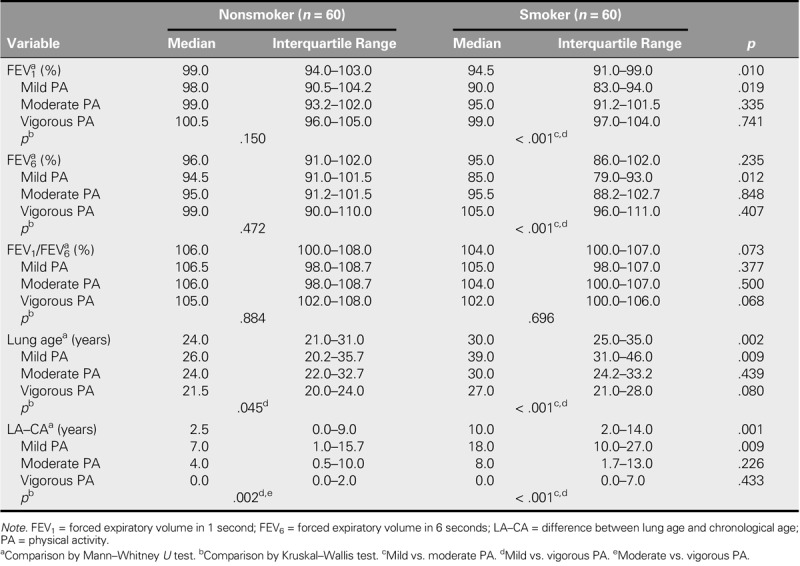
Comparative Study Between the Groups, Stratified According to PA Level Regarding Spirometry Variables

Table 4 shows the assessment results of the multivariate logistic regression models for the association between smoking and PA levels with spirometric variables. With regard to the unadjusted model, being a smoker was associated with worse FEV_1_% (*OR* = 2.72, 95% CI [1.11, 6.69]). Nevertheless, FEV_1_% (*OR =* 0.32, 95% CI [0.12, 0.87]) and FEV_6_% (*OR =* 0.33, 95% CI [0.12, 0.88]) for moderate PA and FEV_1_% (*OR =* 0.18, 95% CI [0.04, 0.67]), FEV_6_% (*OR =* 0.29, 95% CI [0.08, 0.95]), lung age (*OR =* 0.17, 95% CI [0.04, 0.65]), and LA–CA (*OR =* 0.07, 95% CI [0.01, 0.30]) for vigorous PA were shown to be protective factors for pulmonary function. Finally, according to Model 1, smoking was not associated with any of the spirometry variables analyzed. In contrast, moderate and vigorous PA were shown to be a protective factor for pulmonary function outcomes, except for FEV_1_%/FEV_6_%.

**TABLE 4.**
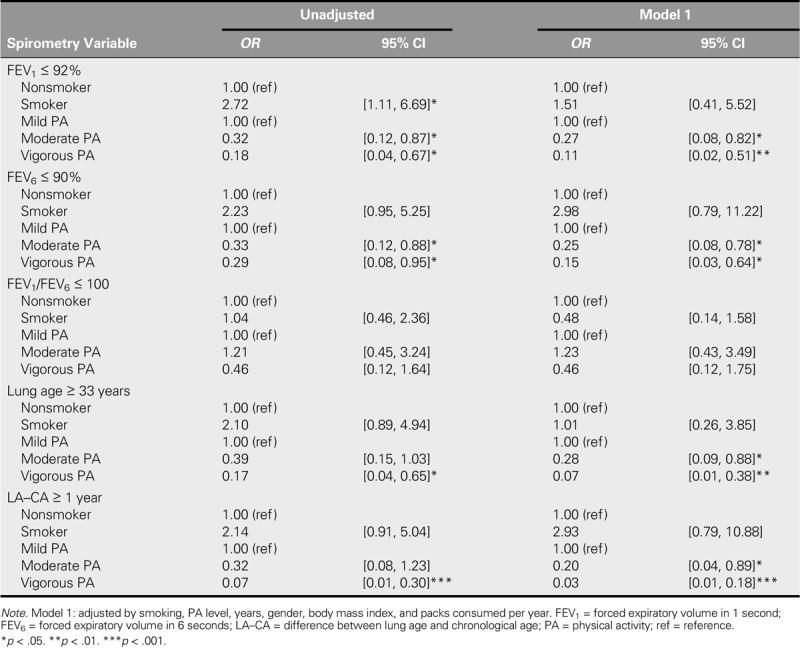
Multivariate Logistic Regression Model of the Association Between Smoking and PA Levels With Spirometry Variables

Table 5 shows the results for the stratified multivariate analysis that was carried out between smokers and nonsmokers. In the context of both models (unadjusted and adjusted), for the smoking group, moderate and vigorous levels of PA were identified as protective factors for the lung function outcomes FEV_1_, FEV_6_, lung age, and LA–CA. However, for the nonsmoking group, only vigorous PA was identified as a protective factor, for LA–CA ≥ 1 year.

**TABLE 5.**
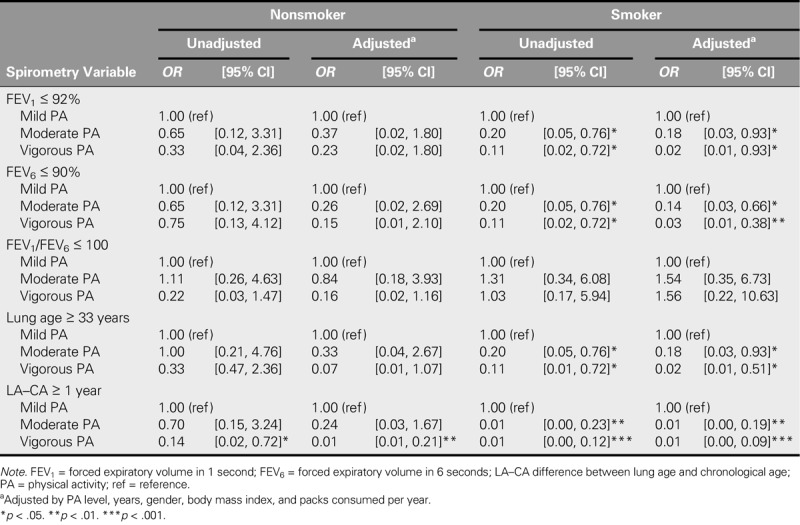
Multivariate Logistic Regression Model of the Association Between Smoking and PA Levels With Spirometry Variables Stratified Into Smoker and Nonsmoker Groups

## Discussion

This study found a beneficial effect for moderate and vigorous PA on pulmonary function (FEV_1_%, FEV_6_%, lung age, and age difference) in a population of nursing students who smoked. There were no significant differences in lung function values between smoker and nonsmoker groups for those participants who practiced moderate or vigorous PA. However, for those participants who practiced mild PA, no beneficial effect was found on pulmonary function in the smoker group, which showed lower spirometric values for FEV_1_% and FEV_6_% and higher lung age and LA–CA.

Spirometry is a basic test for the study of pulmonary function and is necessary for evaluating and monitoring respiratory diseases (García-Río et al., 2013). The quality of this test may be affected by the difficulties faced in correctly obtaining the forced vital capacity (FVC). Therefore, the FEV_6_ has been accepted as an alternative parameter (Jing, Huang, Cui, Xu, & Shen, 2009). This substitution simplifies the spirometry technique, improving accuracy in the diagnosis of airway obstruction in nonspecialized environments (López-Campos, Soriano, & Calle, 2014). In recent years, several portable spirometers have been designed and marketed to obtain FEV_1_, FEV_6_, and their quotient, which, because of their easy handling and cost-effectiveness, are increasingly being used in primary care (Derom et al., 2008). The COPD-6 device is a valid screening tool for COPD and may be used by personnel who have received a brief training session (Represas-Represas et al., 2016). The diagnostic utility of this device has been validated (Represas Represas et al., 2010), and the device has been recently used in several studies to determine pulmonary function (Figueira Gonçalves et al., 2017; Kjeldgaard et al., 2017).

Respiratory health problems related to smoking do not usually manifest fully until later in life, and it is generally assumed that young smokers may not have respiratory problems because of the brief period in which they have been exposed to tobacco smoke. However, the results of this study found that, despite the relatively young age of the participants, the smokers exhibited decreased lung capacity and those that performed only mild PA exhibited significantly decreased lung capacity. Specifically, this was found in the variables FEV_1_%, FEV_6_%, lung age, and LA–CA. Exposure to tobacco smoke throughout life affects pulmonary function and health (Martinez, 2016), with effects varying based on type of smoke exposure and the stage at which individuals are exposed to smoke (Kuh & Ben-Shlomon, 2004). The first years of life represent a critical period of development (Shaheen, 1997) when exposure to smoke may significantly affect the development of pulmonary functions. Furthermore, whereas smoking during adulthood is classically associated with an accelerated decrease in FEV_1_ (Allinson et al., 2016), smoking during adolescence may interfere with the final stages of lung development that influence both FEV_1_ and FVC (Gold et al., 1996). Several studies conducted in young smokers have shown a decrease in lung capacity compared with nonsmokers. Merghany et al. studied 153 male adolescents aged 9–14 years in Khartoum, Sudan, and found that FEV_1_ and FVC were significantly lower in the group that was exposed to tobacco smoke than in the control, nonsmoking group (Merghani & Saeed, 2013). Similar results (in FEV_1_, FEV_1_/FVC, and FEV_25%–75%_) were observed by Bird and Staines-Orozco (2016) in a population of 300 students (137 boys) aged 13–15 years in Juárez, Mexico. Young nonsmokers exhibited significantly higher values than both passive and active smokers. Vianna et al. studied the effects of smoking on the pulmonary function of 2,063 young people with an average age of 24 years in Brazil and found a significant association between smoking and the FEV_1_/FVC ratio (Vianna et al., 2008). In Spain, a similar study was conducted with 2,647 young people (1,275 men) with an average age of 32 years (Urrutia et al., 2005). The authors reported significantly lower values of FEV_1_, FEV_1_/FVC, and FEF_25%–75%_ among young smokers. However, none of the studies mentioned considered PA as a factor of influence on pulmonary function in young people.

Previous studies have reported a positive relationship between the performance of physical exercise and spirometric parameters (Gimeno-Santos et al., 2014; Nystad, Samuelsen, Nafstad, & Langhammer, 2006; Paulo et al., 2013), although other studies have not found a significant relationship (Smith et al., 2016). In addition, the simultaneous effect of PA and smoking on pulmonary function in young people remains unclear. Holmen et al. studied this relationship in 6,811 students aged 13–18 years, observing a significant association between lung capacity (FVC and FEV_1_) and level of physical exercise in those who have never smoked, but not in daily smokers (Holmen et al., 2002). Michalak et al. evaluated the influence of regular PA for 10 months in young smokers and nonsmokers aged 19–24 years, observing improvements in both the FVC and FEV_1_ of both groups (Michalak et al., 2012). Campbell Jenkins et al. studied the relationship between physical inactivity and smoking in pulmonary function in a cohort of 5,301 African American adults aged 21–95 years, concluding that physically active smokers had higher pulmonary functions than the sedentary ones (Campbell Jenkins et al., 2014). This study observed that pulmonary capacity values (FEV_1_%, FEV_6_%, lung age, and LA–CA) improved in the smoker group when the intensity of the PA increased. However, in the nonsmoker group, improvements were only seen in the values for LA–CA. These findings suggest that moderate or vigorous PA may contribute to reducing smoking-related damage to pulmonary capacity.

This study is affected by several limitations. The design was cross-sectional and thus addresses associations only, not causality. In addition, this study used a convenience sample of first-, second-, and third-year nursing students from a university campus in Cáceres, Spain, who were included randomly depending on whether they smoked or not. This is the reason why the numbers of subjects classified into PA categories vary widely. Consequently, the participants may not be representative of university students in other degree programs or even in the same age range, suggesting that the findings may not be applicable to other populations. However, improving the health status of future health professionals was a primary motivation for this work. All of the participants were informed of their spirometric results, including lung age, as there is evidence that knowledge of lung age is a factor that motivates individuals to quit smoking (Ferguson, Enright, Buist, & Higgins, 2000). Furthermore, it has been shown that smoking among health professionals is an important factor affecting the attitudes of society toward this habit (Russell, Wilson, Taylor, & Baker, 1979). Improving the behavior of nursing professionals, as examples and models for the community, may be crucial to achieving behavioral changes in the general population (Aucoin, 1986; Kelly, Wills, & Sykes, 2017). A particular strength of this study was its use, as suggested by Campbell Jenkins et al. (2014), of different categories to assess PA to clarify its effects on lung function. In addition, the young age of the participants may reflect the initial changes in lung function because of tobacco use and the possible benefits of PA. All of these favor an early approach to discouraging smoking.

In conclusion, although the smokers who were assessed in this study were young in age and short-term smokers, the findings confirm that smokers have decreased pulmonary function, as compared with their nonsmoking peers, when they do not regularly perform moderate or vigorous PA. The findings of this study support that level of PA performed is positively related to pulmonary function in smokers, whereas in nonsmokers, the improvements were only significant in LA–CA.
